# Incidence of venous thromboembolism in France: a retrospective analysis of a national insurance claims database

**DOI:** 10.1186/s12959-016-0078-0

**Published:** 2016-02-19

**Authors:** Stéphane Bouée, Corinne Emery, Adeline Samson, Julie Gourmelen, Cécile Bailly, François-Emery Cotté

**Affiliations:** Cemka, 43 bd du Maréchal Joffre, 92340 Bourg-la-Reine, France; Paris-Dauphine University, Paris, France; Bristol-Myers Squibb, Rueil-Malmaison, France; INSERM UMS 011, Villejuif, France

**Keywords:** Incidence, Deep vein thrombosis, Pulmonary embolism, Recurrence, France

## Abstract

**Background:**

Data estimating the annual incidence of venous thromboembolism (VTE) in France, taking into account both hospital and community settings, are very lacking.

This study aimed to estimate the annual incidence of VTE (pulmonary embolism (PE) and deep vein thrombosis (DVT)) in France in 2011 in “real world” population.

**Methods:**

This was a longitudinal insurance claims study of the incidence of VTE in France over 2 years (2010 and 2011). The data analysis was performed using the EGB (*Echantillon Généraliste des Bénéficiaires*) database, a randomly selected sample of the French national insurance database (CNAMTS) which covers 77 % of the population. All adult patients experiencing a VTE event during the study period were analysed. Recurrence rate of VTE and all-cause mortality rate were also estimated over a 12-month follow-up period.

**Results:**

The estimated annual incidence of VTE in France was 184.0 *per* 100 000 subjects, corresponding to a total of 119 670 events countrywide. The estimated incidence of DVT and PE were respectively 119.8 and 64.2 *per* 100 000 subjects. Annual recurrence of VTE was reported in 5.5 % (*n* = 99) patients, with a significantly higher recurrence rate in patients with PE than those with DVT (*p* = 0.02). Overall, 6.2 % (*n* = 112) of patients had died over the 12-month follow-up (respectively 10.2 and 7.7 % of patients with DVT and PE).

**Discussion:**

To our knowledge, this analysis is the first to estimate the annual incidence of VTE in France using exhaustive data from the EGB database. This has allowed the incidence of DVT in the community to be documented, which to date has not been characterised. Previous studies in France have been limited to the hospital setting and have yielded incidence rates comparable to ours.

**Conclusions:**

This analysis is the first to estimate the annual incidence of VTE in France using exhaustive data from the EGB database. This study showed that the incidence and the burden of the disease remains elevated.

## Background

Venous thromboembolism (VTE) is a common condition presenting as deep vein thrombosis (DVT) or pulmonary embolism (PE). DVT of the lower limbs is a potentially life-threatening disease which may be asymptomatic or symptomatic (leg pain, swelling). PE often follows previously asymptomatic DVT and may be revealed by breathlessness, faintness, chest pain, collapse or sudden death. In addition to these short term consequences, long term complications of VTE exist including post-thrombotic syndrome and pulmonary hypertension [1]. Timely detection and management of VTE is important due to the clinically silent nature of VTE in around 80 % of patients [2], its high prevalence in at-risk patients and its potentially life-threatening nature. In a large, population-based survey performed in the US, the mortality rate in hospitalised patients experiencing a VTE was 12 % [3].

VTE is considered as the third most common cardiovascular condition after myocardial infarction and stroke and is a growing public health problem due largely to the ageing population [1]. In a multinational survey performed in 32 countries around the world and enrolling 68 183 patients [4], more than half (51.8 %) of hospitalised patients aged 40 years or over were judged to be at risk for VTE. In North America and Europe, the annual incidence of VTE is estimated to be approximately 160 per 100,000 for DVT, 20 per 100,000 for symptomatic non-fatal PE and 50 per 100,000 for fatal autopsy-detected PE [5]. In France, the incidence of VTE has been estimated in community-based study in Western Brittany performed in 1998–1999 [6] and, more recently, another nationwide study has estimated the incidence of VTE in hospitalised patients to 186.6 per 100 000 subjects [7].

Recently, a number of novel oral anticoagulant agents (NOACs) have been approved for the treatment of VTE. In the context of the introduction of these new therapies, it is important to collect up-to-date and comprehensive data on the burden of VTE in France. This information will be important in order to evaluate the cost-effectiveness of such treatments. To our knowledge, no studies have been published in the last decade that have estimated the incidence of VTE in France, taking into account both hospital and community settings. Recently, a number of public health insurance databases have become available in France which have made it possible to conduct longitudinal nationwide epidemiological and economic analyses providing quasi-exhaustive information on healthcare resource utilisation. This provides an opportunity to reassess the incidence, mortality and morbidity of VTE at a national level. The sponsor of the present study, who markets a NOAC (apixaban) in France, was required by the local regulatory authorities to provide information on the size of the potential target population before introduction of this drug. For this reason, we conducted an analysis of VTE events documented in the EGB (*Echantillon Généraliste des Bénéficiaires)* database, a representative sample of the French national insurance which covers more than three-quarters of the French population [8].

The objectives of this study were to estimate the annual incidence rate of primary VTE (PE and DVT) in 2011 in France in both hospital and community care settings, and to estimate the 12-month incidence rate of recurrent VTE events.

## Methods

This was a longitudinal insurance claims study of the incidence of VTE in France over 2 years (2010 and 2011).

### Data source

The study used data provided by the French health insurance fund for salaried workers (*Caisse Nationale d’Assurance Maladie des Travailleurs Salariés* (CNAMTS)). The CNAMTS database includes all salaried workers and their relatives and covers 77 % of the French population in 2011 (almost 50 million people). Individuals remain covered by the same fund if they stop working for any reason, including retirement, unemployment, invalidity or long-term sick leave. The self-employed, civil servants, students and agricultural workers are the principal socio-professional groups not insured by the CNAMTS. This database contains comprehensive reimbursement records which documents all items of medical consumption in the public or private sectors eligible for reimbursement by public health insurance [8].

The data analysis was performed using the EGB database, which represents a permanent sample of individuals selected at random from 1/97^th^ of all beneficiaries of the CNAMTS and provides data of around 600 000 individuals representative of the French population. This database was developed in 2005 in order to facilitate the conduct of longitudinal epidemiological and economic analyses which are important for planning of healthcare resource attribution [9]. The EGB database contains anonymous information on demographics (age and gender). All eligible medical expenditure reimbursed for a given individual is linked through a unique patient identifier. Items which are not eligible for reimbursement, such as OTC drugs, are absent from the database and cannot be identified. For each prescribed or reimbursed service, the date of implementation is specified, together with the date of prescription and the healthcare provider. No explicit information is provided for the reason for which the service was prescribed, for example the diagnosis. However, in the case of hospitalisations, and only in this case, the diagnosis can be identified since each hospital stay is valued on the basis of a unique diagnosis-related group (DRG) which is coded using the international classification of disease (ICD-10) codes. The reasons for hospitalisation are coded either as primary diagnoses (the condition for which the patient was hospitalised) or as associated diagnoses (comorbidities which may affect the course of hospitalisation). Information on deaths is available in the database but the cause of death is not documented [9]. The EGB database is updated every month.

### Identification of patients with DVT or PE

The target population of the present study consisted of all adult patients with PE or DVT identified in both hospital and community care between 2010 and 2011 documented in the EGB database. For hospitalised patients, patients with PE or DVT were identified from the ICD-10 codes used to assign the DRG associated with the hospital stay (either as a primary or associated diagnosis). The ICD-10 codes used to assign diagnosis were I80.1-9 or I82.1-9 for DVT and I26.0 or I26.9 for PE.

In community care, it was not possible to identify patients with DVT or PE directly from the EGB database since there is no information on diagnosis. For this reason, we constructed an algorithm to identify these patients with DVT from the medical services used. The algorithm for community DVT takes into account three criteria:

1. Reimbursement of an Echo-Doppler exam (day 0: T0)
2. Delivery of a LMWH or fondaparinux (T0 ± 7 days)
3. Delivery of a subsequent Vitamin K antagonists (T0 + 7 days)

Subjects were considered to have a DVT if they fulfilled all these criteria in the above order.

We assumed that all subjects with a confirmed PE were immediately hospitalised and were thus captured through the hospitalisation records.

### Data collection

From the EGB database, we collected the date of all VTE events (PE or DVT) occurring between 2010 and 2011. For each patient, this date was considered to be the index date. The age and gender of each patient was documented. A retrospective analysis of all reimbursement claims over the 12 months preceding the index date was performed. From this data, information on the presence of risk factors for DVT such as orthopaedic surgery and comorbidities were extracted. In addition, reimbursement claims were also evaluated in the 12 months following the index VTE to document all pertinent medical events. These included recurrence of DVT in the community, (defined by the algorithm), hospitalisation for PE, DVT, myocardial infarction, ischaemic stroke, haemorrhage, pregnancy, post-thrombotic syndrome, arterial and pulmonary hypertension. All-cause hospitalisation and death during the 12-month follow-up period were also documented.

### Estimation of incidence

The analysable population consisted of all adult patients experiencing a VTE event during the study period. Study variables were described in the total patient population. All VTE events occurring during the 2-year evaluation period was considered for the estimation of incidence. If an episode of DVT evolved into a PE (clot migration), then the event was considered as an index PE only. Patients who were diagnosed with a VTE in the community and subsequently hospitalised were considered as hospitalised with respect to the index VTE. The incidence of VTE was estimated using EGB database and then extrapolated to the whole French population in 2011 (65 026 885 individuals [10]).

### Data analysis

Continuous data were expressed as mean ± standard deviation and median [range: min-max]. Categorical data were presented as frequency counts (%) with their 95 % confidence intervals.

Incidence of recurrent VTE events (PE and DVT) and other events such as post-thrombotic syndrome, pulmonary hypertension, myocardial infarction, ischemic stroke and bleedings were defined as frequency rates of patients experiencing a new event during the 12-month follow-up period consecutive to the diagnosis of the primary VTE. The cumulative incidence of recurrence of VTE was estimated using Kaplan-Meier survival analysis. The mortality rate was also estimated in this way.

A regression analysis (Cox model) was performed in order to identify factors associated with recurrence of VTE. In a first step, a list of possible factors was identified, including demographics, hospitalisation in the past 3 months, trauma, comorbidities (cardiovascular disease, tumours, renal disease), gastro-intestinal haemorrhage and obesity. Each variable was first evaluated independently in a univariate analysis. In a next step, all factors correlated with recurrence of VTE (at a probability level of 0.05) in the univariate analysis were entered into a multivariate regression analysis in order to identify those independently associated with recurrence of VTE at a probability level of 0.05. In addition, a regression logistic analysis was also performed in order to identify factors associated with hospitalisation for DVT. All identified factors in the univariate analysis were then entered into a multiple regression logistic analysis in order to identify those independently associated with hospitalisation for DVT at a probability level of 0.05. The variables finally retained were entered into a multivariate model to generate odd ratios.

### Ethics

The study was performed in accordance with the International Society for Pharmacoepidemiology (ISPE) Guidelines for Good Pharmacoepidemiology Practices (GPP) and applicable regulatory requirements. Since data was not nominative and was collected retrospectively, ethics board approval was not required. Use of the EGB database was approved by the French national data protection agency (CNIL).

## Results

### Incidence of VTE

The number of VTE events recorded in the EGB database between 2010 and 2011, and the corresponding incidence are presented in Table 1.

**Table 1:** Incidence of VTE in France between 2010 and 2011

Type of VTE event	Number of VTE events in the EGB^a^	Annual incidence (/100 000 subjects; [CI 95 %])	Extrapolation to French population (number of events)
PE	675	64.2 [57.4–71.1]	41 767
DVT (hospital)	673	64.0 [57.2–70.9]	41 643
DVT (comm-care)	586	55.8 [49.4–62.1]	36 260

^a^One patient can have ≥ 1 VTE event

The estimated annual incidence of VTE was 184.0 *per* 100 000 subjects, which corresponds to an estimated annual number of 119 670 events in France. The estimated incidence of DVT was 119.8 *per* 100 000 subjects, corresponding to an annual number of 77 903 events. With respect to PE, the estimated incidence was 64.2 *per* 100 000 subjects, which corresponds to a total of 41 767 events in France. The incidence of both DVT and PE in hospital environment is similar (Table 1).

### Characteristics of the study population

Over the study period (2010 and 2011), a total of 1804 patients with VTE were identified in the EGB database and these constituted the analysis population. Overall, a DVT was identified in 1182 (65.5 %) patients, with approximately equal numbers of patients identified in hospitals (633 patients; 53.5 %) and in community care (549 patients; 46.6 %) (Table 2). Pulmonary embolism was identified in 622 (34.5 %) patients. It was possible to identify the localisation of DVT for 334 hospitalised patients and this was distal DVT in the majority of cases (283; 84.7 % patients).

**Table 2:** Demographics and medical history of the study population

	Patients with PE	Patients with DVT		Total
		Hospital	Community care	
Number of patients (N, (%))	622 (34.5 %)	633 (35.1 %)	549 (30.4 %)	1804 (100 %)
Gender (% women)	57.3 %	69.6 %	54.5 %	57.2 %
Age (years)				
Mean ± SD	67.0 ± 17.2	67.5 ± 17.7	60.3 ± 16.9	65.1 ± 17.6
Median [Min-Max]	71 [19–99]	71 [18–99]	61 [21–94]	68 [18–99]
At least one risk factor of VTE (N, (%))				
Yes	320 (51.4 %)	338 (53.4 %)	159 (29.0 %)	817 (45.3 %)
No	302 (48.6 %)	295 (46.6 %)	390 (71.0 %)	987 (54.7 %)
Principal risk factors (N, (%))				
Hip fracture	2 (0.3 %)	2 (0.3 %)	1 (0.2 %)	5 (0.3 %)
Pregnancy	9 (1.4 %)	7 (1.1 %)	3 (0.5 %)	19 (1.1 %)
Surgery	26 (4.2 %)	24 (3.8 %)	19 (3.5 %)	69 (3.8 %)
Trauma	41 (6.6 %)	54 (8.5 %)	17 (3.1 %)	112 (6.2 %)
Hospitalisation	92 (14.8 %)	112 (17.7 %)	40 (7.3 %)	244 (13.5 %)
Hormonal therapy	54 (8.7 %)	37 (5.8 %)	58 (10.6 %)	149 (8.3 %)
Cancer	129 (20.7 %)	135 (21.3 %)	43 (7.8 %)	307 (17.0 %)
Obesity	31 (5.0 %)	44 (7.0 %)	18 (3.3 %)	93 (5.2 %)

The characteristics of the study population are presented by setting in Table 2. The distribution of the study population with respect to gender is comparable between patients with PE and those with DVT, with a slight but not significant over-representation of women. The mean age was 65 years. Hospitalised patients with DVT or PE were older than patients with DVT in community care (*p* < 0.0001). Overall, 13.5 % of patients had been hospitalised in the previous year. Around half of the study population present at least one risk factor for VTE, most frequently cancer. Hormonal therapy was prescribed in nearly ten percent of patients with DVT or PE.

Among patients with DVT identified in the community care setting (*n* = 549), echography, heparin therapy and VKA were principally prescribed by general practitioners (78.9, 87.0 and 88.4 % of cases respectively). Prescriptions by hospital-based physicians concerned <7 % of patients (5.3, 6.6 and 6.0 % of cases respectively).

### Recurrence of VTE

During the 1-year follow-up of patients identified with an index VTE (*n* = 1804 patients), a total of 99 patients (5.5 %; [95 % CI: 4.5–6.6 %]) experienced a recurrent VTE, most frequently a PE (*n* = 47 patients; 47.5 %) (Table 3). Recurrence of PE was significantly higher than recurrence of DVT (*p* = 0.02; χ2 test). Recurrence of DVT over the follow-up period was similar irrespective of the treatment setting (hospital or community care). In the majority of cases, only one recurrence of the index event (PE or DVT) was identified during the follow-up period.

**Table 3:** Recurrence of VTE during 1-year follow-up period

	PE	DVT		Total	*p* value
		Hospital	Community-care		
At least one recurrence	*N* = 622	*N* = 633	*N* = 549	*N* = 1804	
Yes	47 (7.6 %)	28 (4.4 %)	24 (4.4 %)	99 (5.5 %)	0.019*
No	575 (92.4 %)	605 (95.6 %)	525 (95.6 %)	1705 (94.5 %)	
Frequency of recurrence	*N* = 47	*N* = 28	*N* = 24	*N* = 99	
1	42 (89.4 %)	24 (85.7 %)	21 (87.5 %)	87 (87.9 %)	
2	5 (10.6 %)	3 (10.7 %)	3 (12.5 %)	11 (11.1 %)	
3	-	1 (3.6 %)	-	1 (1.0 %)	
Type of recurrence	*N* = 47	*N* = 28	*N* = 24	*N* = 99	
PE	38 (80.9 %)	4 (14.3 %)	9 (37.5 %)	51 (51.5 %)	<0.0001^#^
DVT (hospital)	9 (19.1 %)	24 (85.7 %)	6 (25.0 %)	39 (39.4 %)	
DVT (comm-care)	-	-	9 (37.5 %)	9 (9.1 %)	

*χ2 test. ^#^Fisher’s exact test

**Fig. 1:**
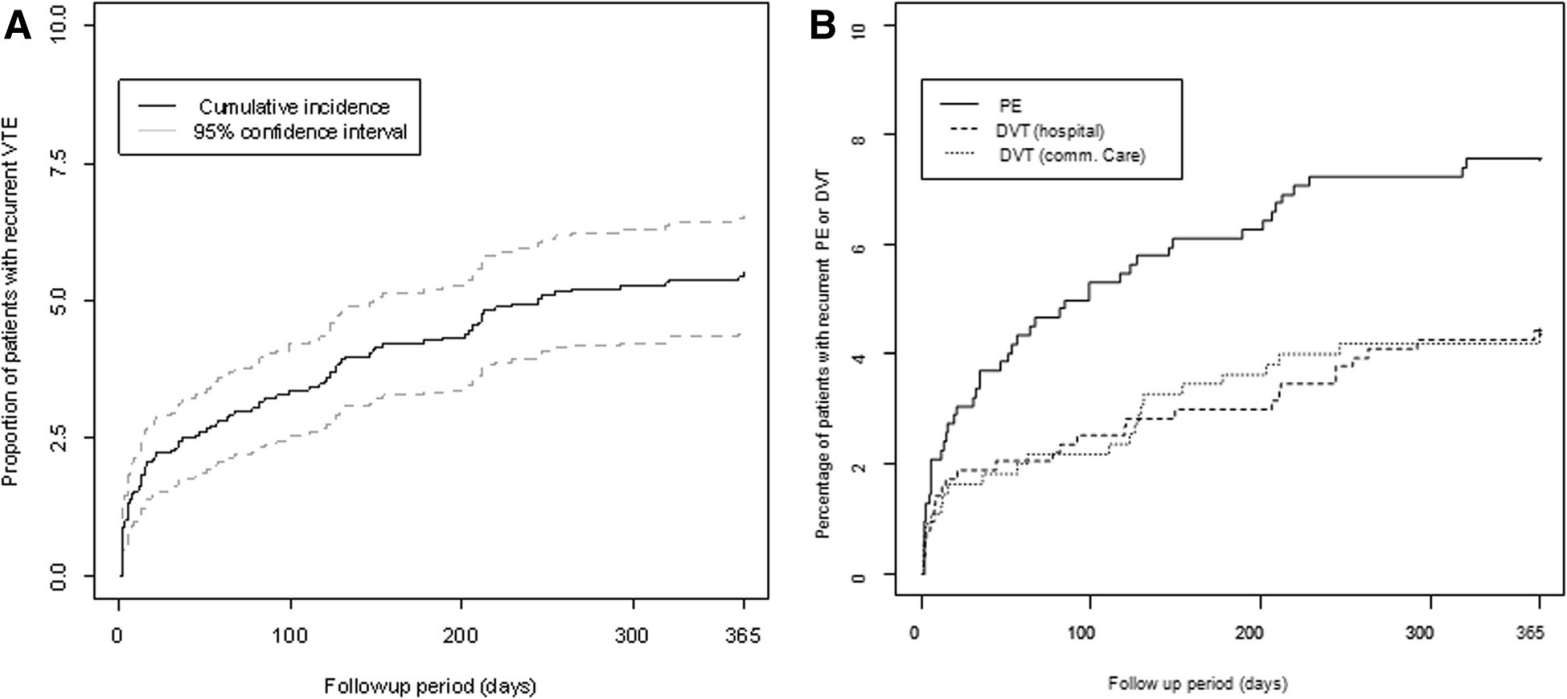
One-year cumulative incidence of recurrences of VTE. **a**: all recurrence events; **b**: recurrence by VTE type

The cumulative incidence of recurrence of VTE during the 1-year follow-up period was estimated at 2.3 % [95 % CI: 1.6–3.0 %] at Month 1, 3.2 % [95 % CI: 2.4–4.0 %] at Month 3, 4.3 % [95 % CI: 3.3–5.2 %] at Month 4 and 5.2 % [95 % CI: 4.2–6.2 %] at Month 9 (Fig. 1a). The time-course of recurrence of DVT was again similar in the two treatment settings (Fig. 1b). The 1-month and 3-month recurrence rates for PE were 3.2 and 5.0 %, and for DVT were 1.9 and 2.4 %.

A regression analysis (Cox model) was performed in order to identify factors associated with recurrence of VTE. The only factor which was correlated with recurrence of VTE during the 1-year follow-up period was hospitalisation for renal failure (data not shown).

### Mortality

**Fig. 2:**
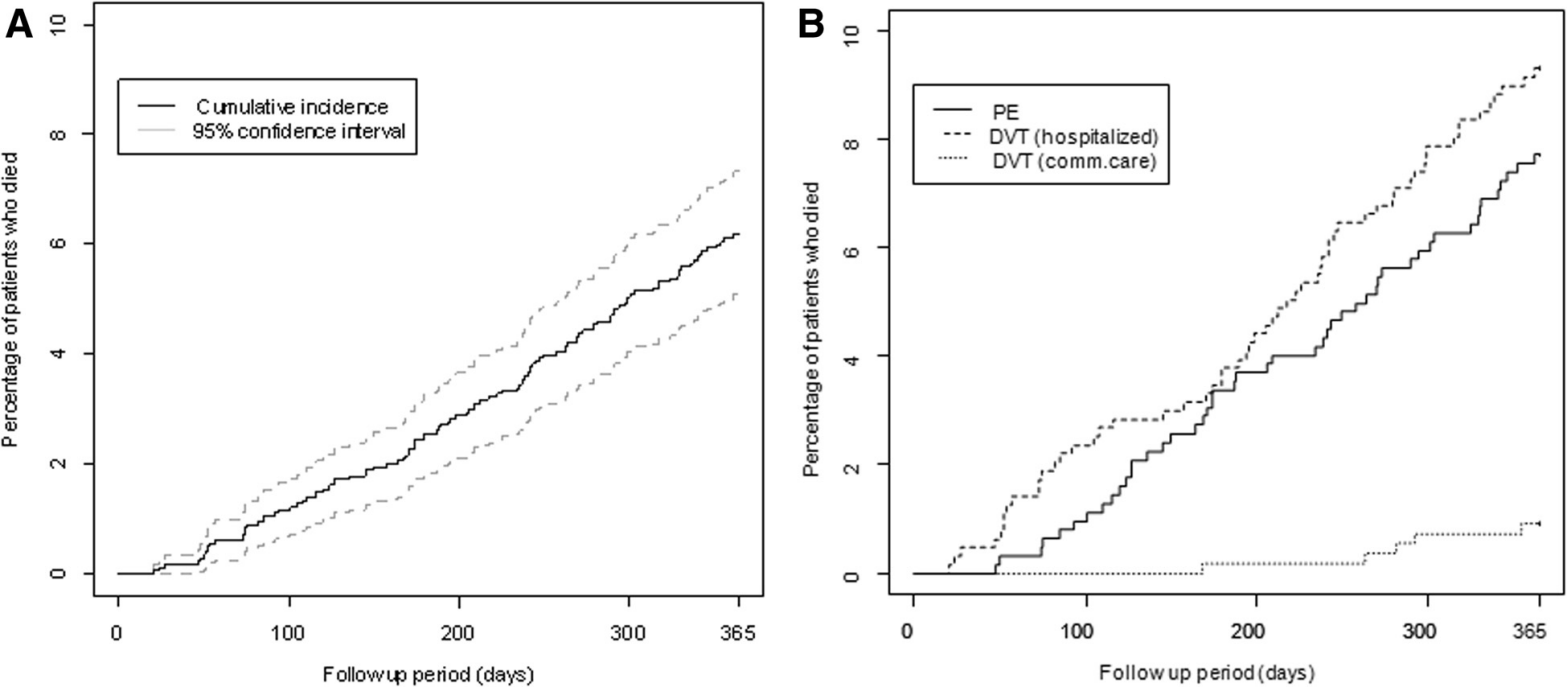
Cumulative incidence of death during the 12-month follow-up **a**: all deaths; **b**: deaths by VTE type

Overall, 6.2 % [95 % CI: 5.1–7.3 %] (*n* = 112) of patients had died by the end of the 12-month follow-up (0.2 % [95 % CI: 0–0.4 %] at Month 1, 1.1 % [95 % CI: 0.6–1.5 %] at Month 3, 2.5 % [95 % CI: 1.8–3.3 %] at Month 6 and 4.3 % [95 % CI: 3.4–5.3 %] at Month 9) (Fig. 2a). The mortality rate was 7.7 % [95 % CI: 5.6–9.8 %] (*n* = 48) in patients with PE, 9.3 % [95 % CI: 7.0–11.6 %] (*n* = 59) in patients with DVT in hospitals and 0.9 % [95 % CI: 0.0–1.7 %] (*n* = 5) in patients with DVT in community care. The mortality rate was significantly higher in hospitalised patients with DVT than those managed in community care (*p* < 0.001) (Fig. 2b).

### Determinants of hospitalisation for a DVT

**Fig. 3:**
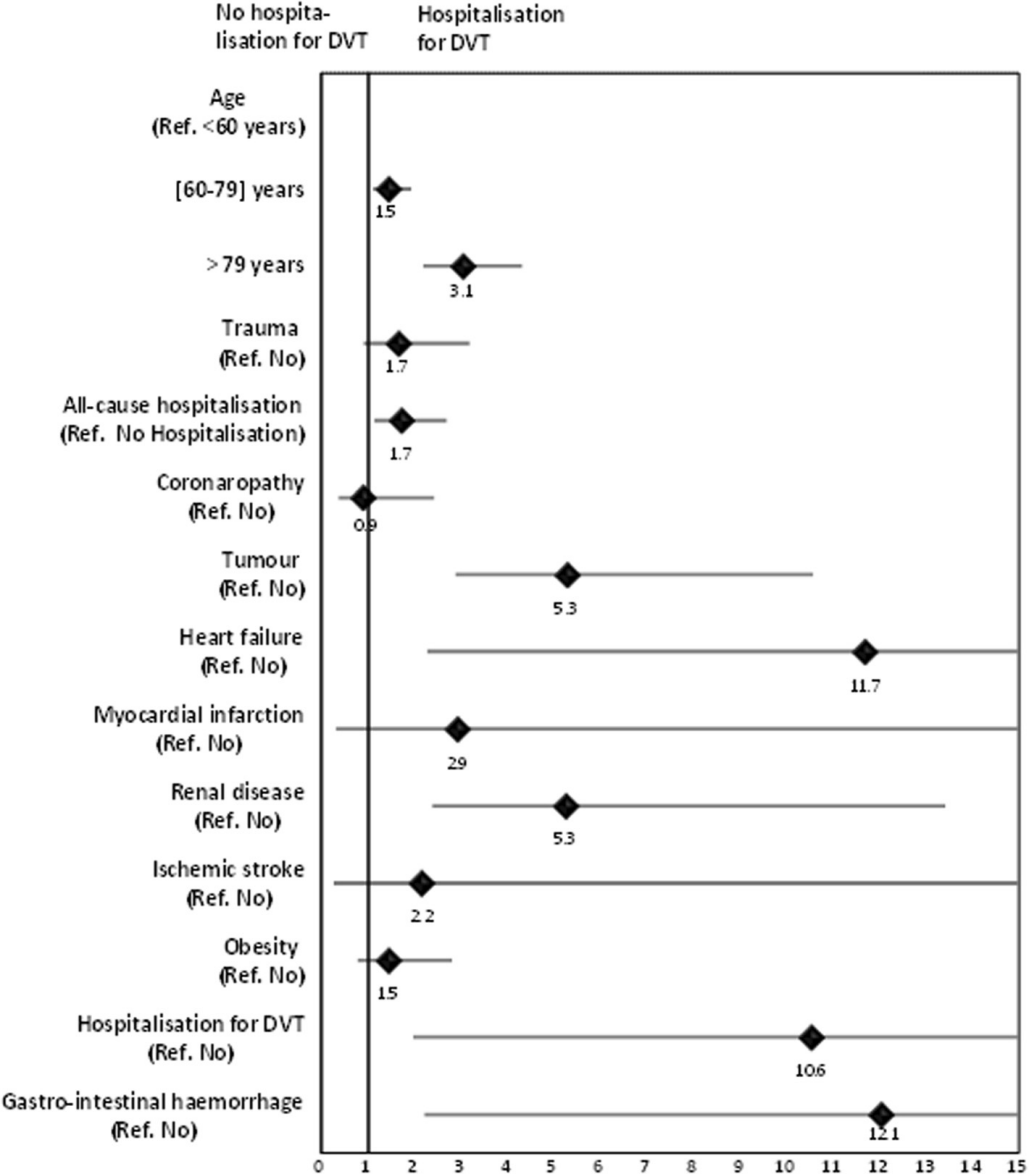
Factors associated with hospitalisation for DVT identified by multiple regression analysis

A regression analysis (logistic model) was performed in order to identify factors associated with hospitalisation for the index DVT. Hospitalisation for DVT was significantly associated with age ≥ 60 years, previous all-cause hospitalisation, presence of tumour, heart failure, renal disease, previous hospitalisation for DVT and gastro-intestinal haemorrhage (Fig. 3).

## Discussion

This retrospective study documented the incidence of VTE (PE and DVT) in France in 2010 and 2011 based on national health insurance claims data. To our knowledge, this analysis is the first to estimate the annual incidence of VTE in France using exhaustive data from the EGB database, allowing the poorly-documented issue of DVT in community care to be addressed. The annual incidence of VTE in France in 2011 was estimated to 184.0 *per* 100 000 subjects. The estimated incidence of DVT was 119.8 *per* 100 000 subjects, corresponding to a total of 77 903 events. The estimated incidence of PE was 64.2 *per* 100 000 subjects, which corresponds to a total of 41 767 events countrywide. It should be noted that since the study was performed from an insurance claims database, patients with VTE who were either undiagnosed or untreated will not be identified.

The estimated incidences of DVT and PE in our study may be compared with previous estimations in France. For example, a community-based epidemiological study performed by a network of hospital and community physicians in Western Brittany in 1998–1999 [6] estimated annual incidence rates of 124 *per* 100 000 subjects for DVT and 60 *per* 100 000 subjects for PE, corresponding to 110 000 patients experiencing a VTE in France per year, of whom 33 % were diagnosed outside the hospital. Other studies in France have been limited to the hospital setting and have yielded incidence rates globally comparable to ours [11]. Notably, the estimated incidence of VTE in hospitals found in our study (128.2 *per* 100 000 subjects), is relatively comparable with the results of a recent study of the national hospitalisation database which estimated the incidence of VTE (as primary diagnoses or as associated diagnoses) in hospitalised patients to 186.6 *per* 100 000 subjects [7].

With respect to demographics, the analysable patients are comparable with the population of the international RIETE registry which includes in 2008 a total of 8 053 patients with symptomatic, objectively confirmed and acute VTE in Spain [12]. Nevertheless, in our study women are over-represented compared to enrolled patients in large and recent randomised clinical trials on VTE [13–16], in which women represented from 42 to 46 % of the total population. This difference may be explained by the fact that overall the proportion of women is admitted to be underrepresented in the majority of randomised clinical trials.

Our study revealed that over 1-year of follow-up, recurrence of VTE was reported in 5.5 % patients. This result is comparable with other estimations in France or in Western countries in which recurrence of VTE occurred in between 5 and 10 % of patients during the first 2-years [17–20]. In addition, data from the RIETE registry including 6361 patients with VTE reported that recurrence of VTE occurred in 3.0 % of patients after 3 months of follow-up [21], which is similar to our estimation (3.2 %). The only factor found to be associated with recurrence was hospitalisation for renal failure. It has been reported in the literature that the risk of recurrent VTE may vary according to age and gender [19, 22, 23], but we were not able to identify any such association in our study.

After 1-year of follow-up, the mortality rate among patients with VTE was estimated in our study at 6.2 %. We found the mortality rate to be higher, without a statistically significant difference, in hospitalised patients with DVT than in patients with PE. This finding was surprising given that PE is generally considered to be associated with a higher recurrence rate and to be potentially most lethal than DVT. One potential explanation for this finding is that PE episodes causing immediate death will not be captured since subjects were not hospitalised or previously diagnosed with PE. It was reported in a study performed in community-care in the UK in 2007 that of 730 fatal cases of VTE, ninety percent related to death on the date of diagnosis of PE [24]. Moreover, the 3-month fatality rate after PE seems to be underestimated (0.8 %) in our study compared to the literature. For example, in the OPTIMEV study the 3-month mortality rate was estimated to be 12.9 % in patients with PE secondary to DVT and 4.6 % in patients with primary PE [25]. Again, this may be attributed to failure to capture immediately fatal VTE events. A striking finding of the study was that the mortality rate after DVT was tenfold higher in hospitalised patients than those managed in community-care environment (9.3 % *versus* 0.9 % respectively). It should be noted that no information on the cause of death is provided in the EGB database, and to what extent the deaths reported can be attributed directly or indirectly to VTE is unknown.

This study also allowed us to assess factors potentially associated with the care setting, which distinguished between patients with DVT managed in the hospital and those managed in community-care. Several such determinants of hospitalisation were identified, including older age, previous all-cause hospitalisation, presence of tumours, heart failure, renal disease, previous hospitalisation for DVT and gastro-intestinal haemorrhage. Taken together, this suggests that hospitals take care of the more fragile patients. These factors are consistent with guidelines from the French Health agency (ANSM) for the management of DVT in hospitals [26]. Nonetheless, around half of the patients with DVT were managed in the community and this proportion is higher than that reported a decade previously in the survey in Brittany (France) [6]. This evolution may reflect better understanding of VTE and its management by community-based physicians and represents potentially important economies for the health service.

This study provided little information about treatment of DVT. By definition, all community DVT patients identified in our study were treated with a heparin followed by a VKA. International and French treatment guidelines for hospitals recommend acute treatment of VTE with heparins followed by pharmacological prophylaxis with VKA to prevent recurrence [26–28]. During the observation period of the study, NOACs had not yet been made available in France. Moreover, in addition to the known risk factors associated with recurrence of VTE such as previous hospitalisation or cardiovascular diseases, recurrence of VTE will also depend on VTE prophylaxis after the index event. For example, in the pivotal studies of new oral anticoagulants, the recurrence rate was somewhat different between treatment groups and ranged from 1.3 to 9.8 % of patients [13, 14, 29, 30].

The incidence of VTE observed in this study may be expected to change over the coming years due to the introduction of new treatments, notably non-VKA oral anticoagulant drugs. These have been shown to reduce the risk of VTE and PE [16], as well as other serious vascular events such as stroke [31] and acute coronary syndrome [32, 33].

The study presents a number of strengths. The most important of these is the exhaustiveness of the data available in the EGB database, which at the time of the study, provided information on a random sample of 77 % of the French population [8]. The EGB sampling method is based on a national identifier attributed to each French resident which allows all health care consumption to be followed until death. However, this database has some limitations for this type of epidemiological study. Data analysis is retrospective, as is usual for insurance claims studies, and this restricts the analysis to information routinely documented in the database. In community care, no information on diagnosis is available and the reasons for prescription of medication and medical procedures are not documented, either in hospital or in community care. In this respect, a notable and novel feature of the study was the design of a specific algorithm to identify patients with DVT in the community from insurance claims records. For hospitalised patients, incomplete or erroneous documentation of DRGs, and thus diagnoses, cannot totally be excluded since there was no possibility for case ascertainment through the medical records. Finally, we made the assumption that all cases of PE were managed in hospitals, but it cannot be excluded that some patients with PE in the community have not been diagnosed correctly. There is thus some uncertainty in our estimations, in particular for DVT events managed out of hospitals. Another limitation is that the EGB database does not capture information about clinical reasons for death and consequently we cannot estimate the specific VTE-related mortality rate in our study.

## Conclusion

Primary VTE (PE and DVT) affects around 120,000 individuals in France each year, of whom one in twenty has a recurrent VTE and one in sixteen dies within the following year. The burden of the disease remains elevated, in spite of effective treatments and better understanding of risk factors and their prevention. The number of patients with DVT managed in the community increases, particularly in the less vulnerable patients, and this is associated with better long-term outcome.
